# Serum *N*-glycan profiles differ for various breast cancer subtypes

**DOI:** 10.1007/s10719-021-10001-3

**Published:** 2021-04-20

**Authors:** Gerda C. M. Vreeker, Kiki M. H. Vangangelt, Marco R. Bladergroen, Simone Nicolardi, Wilma E. Mesker, Manfred Wuhrer, Yuri E. M van der Burgt, Rob A. E. M. Tollenaar

**Affiliations:** 1 Department of Surgery, Leiden University Medical Center, Leiden, The Netherlands; 2 Center for Proteomics and Metabolomics, Leiden University Medical Center, P.O. Box 9600, 2300 RC Leiden, The Netherlands

**Keywords:** Breast cancer, Glycomics, Serum protein glycosylation, Mass spectrometry, Glycan profiling, Tumor heterogeneity

## Abstract

Breast cancer is the most prevalent cancer in women. Early detection of this disease improves survival and therefore population screenings, based on mammography, are performed. However, the sensitivity of this screening modality is not optimal and new screening methods, such as blood tests, are being explored. Most of the analyses that aim for early detection focus on proteins in the bloodstream. In this study, the biomarker potential of total serum *N*-glycosylation analysis was explored with regard to detection of breast cancer. In an age-matched case-control setup serum protein *N*-glycan profiles from 145 breast cancer patients were compared to those from 171 healthy individuals. *N*-glycans were enzymatically released, chemically derivatized to preserve linkage-specificity of sialic acids and characterized by high resolution mass spectrometry. Logistic regression analysis was used to evaluate associations of specific *N*-glycan structures as well as *N*-glycosylation traits with breast cancer. In a case-control comparison three associations were found, namely a lower level of a two triantennary glycans and a higher level of one tetraantennary glycan in cancer patients. Of note, various other *N*-glycomic signatures that had previously been reported were not replicated in the current cohort. It was further evaluated whether the lack of replication of breast cancer *N*-glycomic signatures could be partly explained by the heterogenous character of the disease since the studies performed so far were based on cohorts that included diverging subtypes in different numbers. It was found that serum *N*-glycan profiles differed for the various cancer subtypes that were analyzed in this study.

**Supplementary Information:**

The online version contains supplementary material available at 10.1007/s10719-021-10001-3.

## Introduction

Worldwide 2,089,000 women were diagnosed with breast cancer with an estimated related death of 626,000 in 2018 [1]. Population-based breast cancer screening reduces mortality and is commonly performed with mammography [2]. However, mammography-based screening can be improved with regard sensitivity and specificity levels. It is furthermore known that tumors in dense breast tissue are often missed in a mammogram and although outweighed by mortality reduction low energy X-ray imaging carries a risk of causing radiation-induced tumors [3]. Available clinical biomarkers cancer antigen (CA) 15 − 3, 27–29 and 125 as well as carcinoembryonic antigen (CEA) are only of use to indicate treatment failure and are not recommended for screening, diagnosis, or staging purposes [4]. Therefore, discovery of novel biomarkers with improved test performance is widely pursued to potentially provide an add-on diagnostic tool [5]. Next to genomic markers, proteins that are present in the circulation have received great attention [6, 7]. Although a large number of mass spectrometry (MS)-based exploratory studies has resulted in breast cancer protein signatures, none of these findings has been translated into a laboratory test [8]. As a consequence, biomarker strategies have been improved by properly defining the unmet clinical needs and by implementing protocols for standardized body fluid collection, high-throughput sample preparation and robust and precise MS-measurements [5, 9–12].

At the same time, MS-based proteomics studies demonstrated that post-translation modifications (PTMs) on proteins are often overlooked, although these modulate protein function and are thus an interesting source of functional biomarkers. One of the most, if not the most frequent PTMs is protein glycosylation [13–15]. Changes in protein glycosylation may have influence on or may be caused by tumor growth, differentiation, metastasis, transformation, adhesion, pathogen recognition and immune surveillance [16, 17]. Protein glycosylation and its association with various cancers has been studied for more than half a century, but recent developments have allowed glyco(proteo)mics strategies to join forces with high-throughput cancer proteomics efforts to determine glycomic phenotypes and improve our understanding of the pathophysiology of various cancers [18–24]. For example, large-scale glycosylation biomarker studies based on for example immunoglobulin glycosylation and total serum *N*-glycome (TSNG) have reported changes upon cancer treatment and associations with survival [25, 26]. Moreover, aberrant glycosylation profiles have been found on the surface of cancer cells with potentially diagnostic value towards evaluating tumor progression [27, 28]. Breast cancer biomarker signatures have been pursued by analysis of *N*-glycan profiles in blood-derived or other body fluid samples using ultrahigh performance liquid chromatography (UPLC) methods combined with MS identification or detection of fluorescent labels [29–36]. These studies reported associations with cancer or treatment regimes, but interestingly did not always corroborate previous findings.

In this study we report TSNG profiles from an in-house collected breast cancer cohort and compare our results with the aforementioned reports. Our sample cohort consists of 145 breast cancer patients that are age-matched with 171 healthy control individuals. *N*-glycan analysis includes linkage-specific derivatization of α2-3- and α2-6-linked sialic acids and MS-profiles are obtained using a matrix-assisted laser desorption/ionization Fourier Transform ion cyclotron resonance (MALDI-FT-ICR) platform. The potential of *N*-glycan profiles for diagnosis or staging of breast cancer is evaluated.

## Materials and methods

### Patients

 Serum samples of 159 female patients with breast cancer and 173 female healthy volunteers were collected at the outpatients clinic at Leiden University Medical Center prior to any treatment between 2002 and 2013. The samples of the controls were matched to the cases based on age and date of sample collection. Criteria for case exclusion were; a history of cancer (other than basal cell carcinoma or cervical carcinoma in situ) shorter than 10 years before blood sampling and breast cancer in medical history. From the controls only date of birth was recorded. Table 1 provides an overview of patient characteristics and information on the invasive cancer cases (i.e. excluding ductal carcinoma in situ (DCIS). Written informed consent was obtained from patients and healthy volunteers prior to sample collection. The study was approved by the Medical Ethical Committee of the LUMC.

**Table 1:** Patient characteristics and invasive tumor characteristics

	%	Cases (*n* = 145)	Controls (*n* = 171)
Age in years, mean (SD)		68 (13.1)	67 (11.2)
Histological type			
DCIS	16	23	n/a
Invasive ductal carcinoma	66	96	n/a
Invasive lobular carcinoma	14	21	n/a
Other	4	5	n/a
Invasive tumors (n = 127)			
Grade			
I	18	26	n/a
II	40	58	n/a
III	37	53	n/a
Missing	5	8	n/a
Tumor stage			
pT1	67	80	n/a
pT2	30	36	n/a
pT3/4	3	4	n/a
Nodal stage			
N0	63	77	n/a
Nmi	4	5	n/a
N1	26	32	n/a
N2	3.5	4	n/a
N3	3.5	4	n/a
Estrogen receptor (ER)- status			
Negative	16	23	n/a
Positive	68	98	n/a
Missing	16	24	n/a
Progesterone receptor (PR)-status			
Negative	54	78	n/a
Positive	30	43	n/a
Missing	16	24	n/a
Human epidermal growth factor receptor-2 (Her2)-status			
Negative	68	99	n/a
Positive	10	15	n/a
Missing	22	31	n/a

n, number of individuals; SD, standard deviation; n/a, not applicable;

### Serum sample collection

Blood specimens were collected and processed according to a standardized protocol. Blood was collected in a 8.5 cc vacutainer serum separator tube and centrifuged for 10 min at 1000 g. After centrifugation the serum was divided into 5 mL polystyrene tubes. Within 4 h after blood collection the serum samples were stored at -80 °C. The samples underwent one freeze-thaw cycle for aliquoting in eight 60-µl tubes. All serum samples were randomly distributed in six 96-well plates, along with plasma standards (Visucon-F frozen normal control plasma, pooled from 20 human donors, citrated and buffered with 0.02 M HEPES, Affinity Biologicals, Ancaster, ON, Canada) as technical quality control samples and blanks.

### Chemicals

Nonidet P-40 substitute (NP-40), potassium dihydrogenphosphate (KH_2_PO_4_), disodium hydrogen phosphate dihydrate (Na_2_HPO_4_ × 2 H_2_O), sodium chloride (NaCl), 50 % sodium hydroxide (NaOH), 1-hydroxybenzotriazole 97 % (HOBt) and super-DHB (9:1 mixture of 2,5-dihydroxybenzoic acid and 2-hydroxy-5-methoxybenzoic acid, sDHB) were obtained from Sigma-Aldrich (Steinheim, Germany). Potassium hydroxide (KOH), sodium dodecyl sulfate (SDS), analytical grade ethanol and trifluoroacetic acid (TFA) were purchased from Merck (Darmstadt, Germany). HPLC-grade acetonitrile (ACN) was purchased from Biosolve (Valkenswaard, The Netherlands) and 1-ethyl-3-(3-(dimethylamino)propyl)carbodiimide (EDC) hydrochloride was obtained from Fluorochem (Hadfield, UK). Recombinant peptide-*N*-glycosidase F (PNGase F) was purchased from Roche Diagnostics (Mannheim, Germany). From a Millipore Q-Gard 2 system (Amsterdam, The Netherlands) maintained at ≥ 18 MΩ milli-Q water (MQ) was generated.

### Sample preparation and MALDI-FTICR-MS measurement

Enzymatic *N*-glycan release was performed as previously described [37]. In short, 6 µL sample was added to 12 µL 2 % SDS and incubated for 10 min at 60 °C. After incubation 12.6 µL release mixture (6 µL 4 % NP40, 6 µL 5× PBS and 0.6 µL PNGase F) was added and the samples were incubated overnight at 37 °C. The samples were stored at -20 °C before further preparation.

Ethyl esterification was performed for linkage specific stabilization of the sialic acid moieties of the glycans [38]. One microliter of released glycan sample was added to 20 µL of ethyl esterification reagent (0.25 M EDC 0.25 M HOBt in pure ethanol) and incubated for one hour at 37 °C. Subsequently 20 µL ACN was added.

Glycan purification was performed using cotton HILIC SPE microtips [38, 39]. These HILIC tips were prewetted with three times 20 µL MQ and conditioned with three times 20 µL 85 % ACN. Next, the sample was pipetted up and down 20 times in the HILIC tip. The HILIC phase was first washed three times with 20 µL 85 % ACN with 1 % TFA and second three times with 20 µL 85 % ACN. Elution was performed by pipetting 10 µL MQ five times up and down. Two microliters of sample was spotted with 1 µL matrix (5 mg/mL sDHB in 50 % ACN with 1 mM NaOH) onto a MALDI target (800/384 MTP AnchorChip, Bruker Daltonics, Bremen, Germany) and the spots were allowed to dry.

MALDI-FTICR-MS experiments were performed as described before [40]. A Bruker 15T solariX XR FTICR MS (Bruker Daltonics) recorded the spectra in the *m/z*-range from 1011.86 to 5000.00, with 1 M data points. The obtained average spectra contained ten acquired scans. The system was operated by ftmsControl (version 2.1.0) software.

### Data preprocessing, batch correction and statistics

Serum *N*-glycosylation profiles were obtained for 159 breast cancer patient samples and 173 healthy volunteer samples, of which respectively 145 and 171 spectra passed the quality criteria [40]. The analyte list consisted of 101 analytes which passed the quality criteria (Supporting information Table S-1). The areas of the signals were extracted using MassyTools (version 0.1.8.1). To correct for batch effects from the two MALDI-target batches (number of samples exceed the number of spots on a MALDI-target), the effect was estimated per analyte in a linear model and the values of these analytes were regressed on the MALDI-target batch (categorical variable). The standardized values were normalized to the sum of all analytes for relative quantification. Subsequently, derived traits were calculated (Supporting information Table S-2) and logistic regression was performed for each individual glycan and each derived trait using R version 3.3.2 (R Foundation for Statistical Computing, Vienna, Austria) and RStudio, version 1.0.136 (RStudio, Boston, MA; released 21 December 2016) [41]. The odds ratios (ORs) were calculated with their 95 % confidence intervals (CIs) assuming a Student’s t-distribution and are referring to an increase of 1 SD in the tested traits. Multivariate (principal component) analysis was performed on both individual glycans and derived traits using the various clinical parameters of the breast cancer subtypes.

## Results and discussion

Serum protein *N*-glycan profiles were obtained from an in-house breast cancer cohort, consisting of 145 breast cancer cases and 171 healthy controls. In total 101 *N*-glycans were relatively quantified, including differentiation species with α2-3- and α2-6-linked sialic acids (see Materials and Methods section). Patient characteristics and information on the invasive cancer cases (i.e. excluding ductal carcinoma in situ (DCIS) is provided in Table 1. The patient group had an average age of 68 years and almost half of the group had stage II breast cancer. Quality control samples were taken along in the TSNG analysis to enable potential batch correction, as described in materials and methods.

Logistic regression analysis was performed to reveal potential differences between the glycosylation profiles of breast cancer patients and healthy controls. Moreover, it was evaluated whether glycosylation associated with one of the various clinical parameters listed in Table 1. This was done by using multivariate (principal component) analysis as well as by assuming a *t*-distribution of the various breast cancer subtypes. All analyses were performed for both single compositions and combined glycosylation features (further referred to as derived traits), of which the latter analysis focused on the most commonly reported cancer-associated changes in glycosylation, namely sialylation, fucosylation, and N‑linked glycan branching [30].

**Fig. 1:**
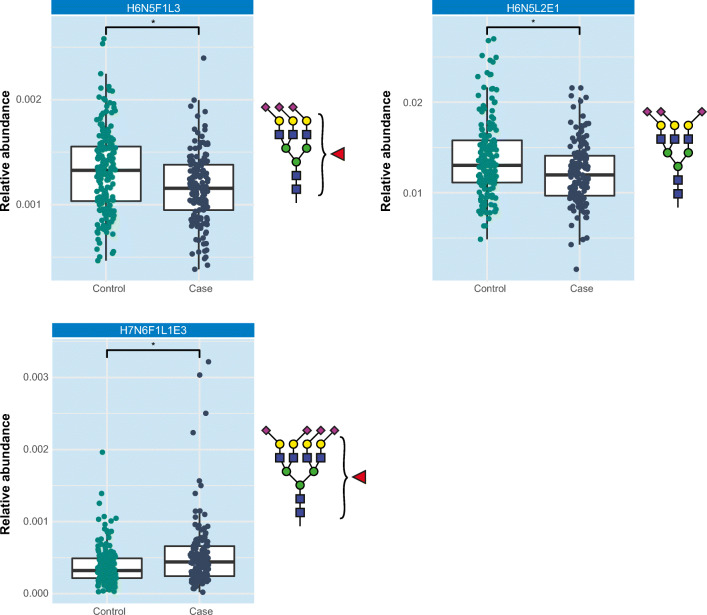
Association of H6N5F1L3, H6N5L2E1 and H7N6F1L1E3 with breast cancer

**Fig. 2:**
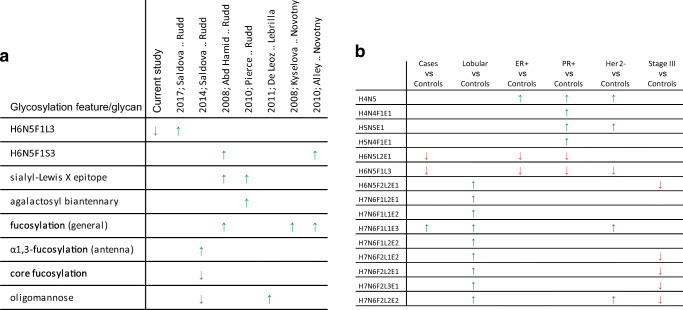
**a** Comparison of previously reported data and results of the current study. **b** Significant direct traits (glycan compositions) for specific breast cancer subtypes and stages as determined in a Student’s t-test

Student’s *t*-test indicated two glycans to be lower in breast cancer patients, namely a fucosylated triantennary glycan that carries three α2-3-linked sialic acids (further referred to as H6N5F1L3, Fig. 1, Supporting information Table S-3 and Supporting material) and a non-fucosylated triantennary glycan that carries a combination of α2-3-linked and α2-6-linked sialic acids (H6N5L2E1). Furthermore, it was found that one fucosylated tetraantennary glycan that carries a combination of α2-3-linked and α2-6-linked sialic acids (H7N6F1L1E3) was significantly elevated in breast cancer patients. Interestingly, in one previous study H6N5F1L3 has been associated with breast cancer, however in the opposite direction with elevated levels in patients as compared to controls (as is summarized in Fig. 2a) [30]. Similar elevated levels of triantennary trisialylated fucosylated glycans were reported in earlier studies, although it is emphasized that in these studies sialic acids were not determined with linkage-specificity, but rather as summarized triantennary trisialylated fucosylated glycans (referred to as H6N5F1S3, consisting of H6N5F1E3, H6N5F1L3, H6N5F1L2E1, H6N5F1L1E2 and H6N5F1E3, Supporting information Table S-3) [32, 42].

In one of the older studies a significant increase was found in trisialylated triantennary glycans containing α1-3-linked fucose, pointing towards elevated levels of the sialyl-Lewis X (sLe^x^) epitope [32]. Similarly, Pierce and co-workers reported elevated levels of agalactosylated diantennary glycans and glycans containing the sLe^x^ epitope in patients with tumor-positive lymph nodes compared to women with no lymph node metastasis [33]. Such increased levels of the sLe^x^ epitope in serum and on the tumor cell surface are frequently associated with cancer [30, 31, 43–47], but were not observed in our study upon considering different stages.

**Fig. 3:**
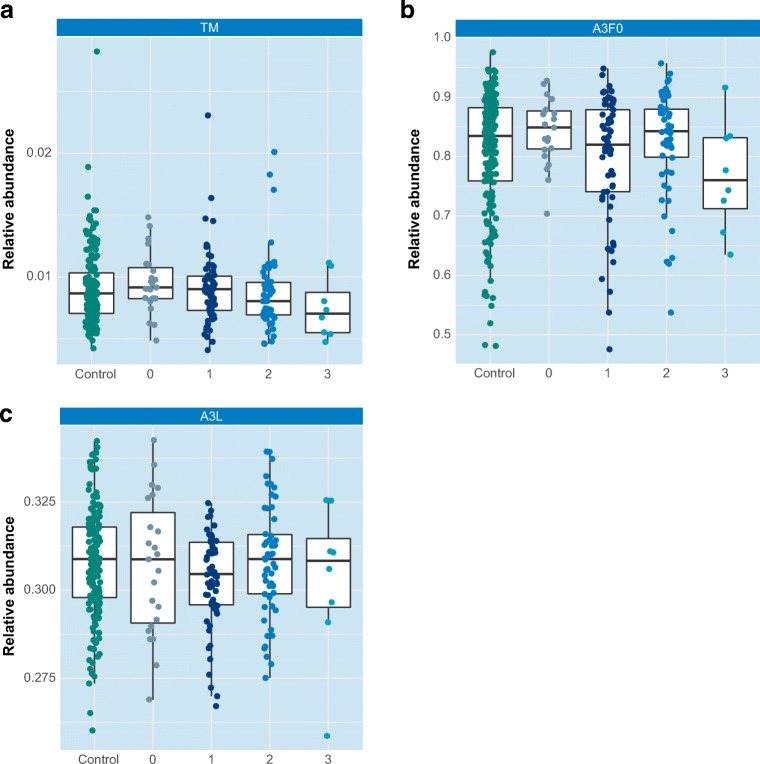
Three examples of derived traits and their potential association with cancer stage. Control individuals are plotted in green, patients with DCIS are plotted in grey (0), breast cancer patients are plotted in blue with staging 1 = grade I, 2 = grade II, 3 = grade III. **a** Oligomannose structures (TM) **b** Non-fucosylated triantennary glycans (A3F0) **c** α2-3-sialylated triantennary glycans (A3L)

With regard to the analysis of derived glycosylation traits from our data, TSNG profiles showed differences for CF, A2LF and A2F0B between breast cancer patients and healthy controls (Supporting information Table S-4). Additional differences were found when clinical parameters (Table 1) were taken into account as summarized in Fig. 2b. Upon considering cancer staging, as an example the levels of oligomannose structures in breast cancer cases are plotted in Fig. 3a. A trend towards a lower level of oligomannose can be seen at stage III cancer, whereas in a previous mouse study on breast cancer elevated levels of oligomannose glycans were observed [34]. In the same study a decreased level was reported after resection and furthermore a small number of case-control human serum samples were evaluated, in which similar elevations of oligomannose glycans were observed in breast cancer patients [34]. In addition, this elevation was supported by a breast cancer cell line study [48]. Here, released glycans from cytosolic and membrane-bound glycoproteins from normal epithelial cells, invasive and non-invasive breast cancer cells were measured with MALDI-MS and the obtained profiles were compared. Notably, a decrease of oligomannose glycans in serum of breast cancer patients has also been reported [31], and literature findings on serum oligomannose glycan levels of total serum appear contradictory.

**Fig. 4:**
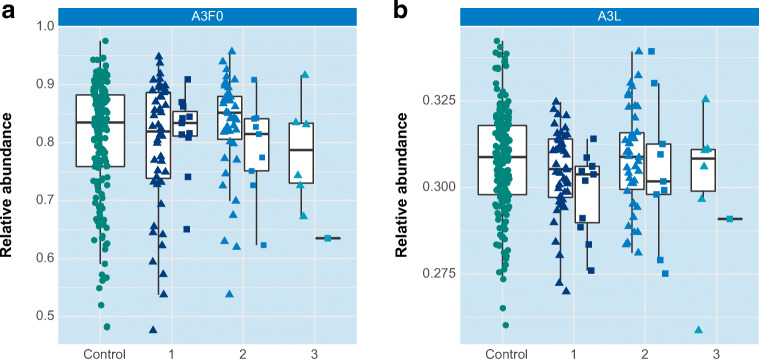
Two examples of derived traits and their potential association with cancer stage. Control individuals are plotted in green, breast cancer patients are plotted in blue with staging 1 = grade I, 2 = grade II, 3 = grade III. Breast cancer patients are further stratified according to histology, namely “Invasive ductal carcinoma” (triangles) and “Invasive lobular carcinoma” (squares) **a** Non-fucosylated triantennary glycans (A3F0) **b** α2-3-sialylated triantennary glycans (A3L)

Results for fucosylation and sialylation traits are exemplified in Fig. 3b (triantennary non-fucosylated glycans; A3F0) and Fig. 3c (α2-3-sialylation of triantennary glycans; A3L), respectively. This data which is obtained from a fair number of patient samples (n = 145) is not in line with previous findings of increased fucosylation and sialylation levels associated with cancer progression and staging of the disease [29, 32, 42]. However, when other clinical parameters are considered certain derived traits exhibit significant p-values, for example when only lobular carcinomas are compared to controls (CF, A3F, A2LF, A3LF, A3EF and A4EF, see Supporting material). Moreover, when considering stage III patients with lobular carcinoma the levels of the three earlier mentioned glycan compositions (Fig. 1) are increased by a factor of 1.5, whereas in stage III patients with ductal carcinoma these levels are decreased by a factor of 2. Although these latter observations are not significant (due to low sample numbers), this is a clear indication that the heterogeneous character of breast cancer that includes a large number of disease subtypes (as summarized in Table 1) is reflected in various *N*-glycan profiles. Of note, for our current data set, stratification according to histological subtypes did result in clear disease glycomic signatures yet. This is exemplified for fucosylation and sialylation in Fig. 4a and b, respectively, where glycomic data are plotted separately for the two histological breast cancer types. No statistically significant were observed, possibly due to limited sample numbers. It is noted that patient cohorts in earlier studies likely consisted of different combinations of these histological subtypes. The various results reported so far emphasize the importance of detailed knowledge of clinical data and inclusion of even larger patient numbers.

In conclusion, we have analyzed serum *N*-glycosylation profiles from breast cancer patients and healthy controls. A distinguishing signature for breast cancer was not found, although a significant difference between both groups were observed for H6N5F1L3, H6N5L2E1 and H7N6F1L1E3. In previous studies, various changes in TSNG were reported, but also these results differed from each other and could not be replicated in our study. An evaluation of literature, together with the results of the current study, does not converge into a general breast cancer N-glycomic signature that distinguishes cases from controls. However, the fact that such glycomic markers are not observed can be explained by the heterogeneity of the disease and by the small size of patient cohorts. The heterogeneous character of the disease becomes clear from Table 1 that lists patients that exhibit various combinations of receptor statuses. Furthermore it is known that breast cancer tumors present a variety of histological patterns and biological characteristics [49]. In addition, the clinical response of breast cancer tumors is very different per type and up to 25 % of the invasive breast cancer tumors is histologically seen a special type [49]. It is therefore recommended that in future biomarker discovery studies different subtypes within the breast cancer samples should be taken into account, instead of analyzing all breast cancer tumor subgroups together and aiming for an overarching signature.

## Supplementary Information

ESM 1 (XLSX 4.75 MB)

ESM 2 (PDF 213 KB)

ESM 3 (PDF 503 KB)

ESM 4 (XLSX 761 KB)
