# Supplementary material for: Serum N-glycan profiles differ for various breast cancer subtypes
Source: Glycoconj J. 2021 Apr 20;38(3):387–95. doi: 10.1007/s10719-021-10001-3 (PMC8116229; doi:10.1007/s10719-021-10001-3)
Supplement: Supplementary file 2 — (PDF 213 KB) [file 10719_2021_10001_MOESM2_ESM.pdf]

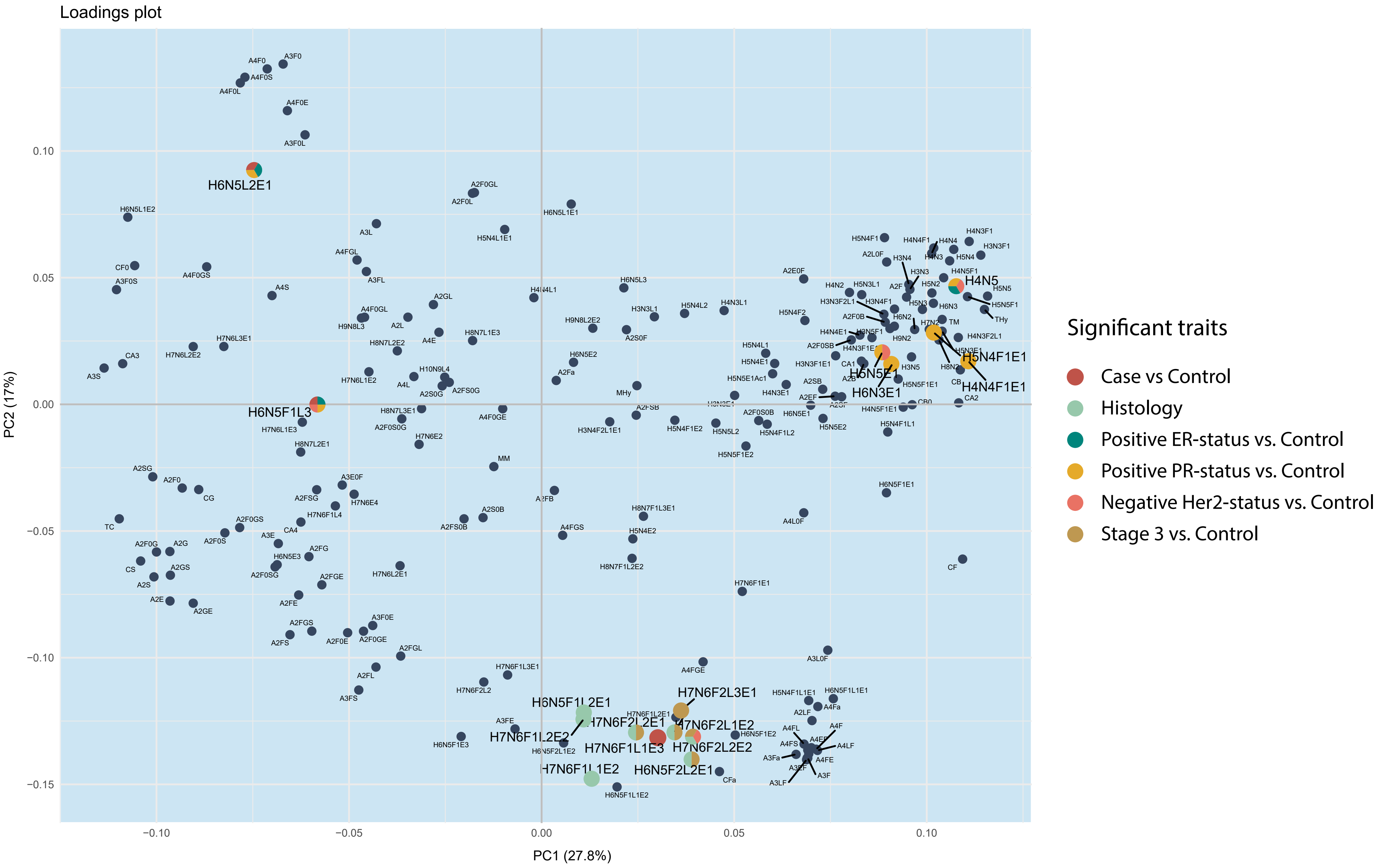
Loadings plot
PC1 (27.8%)
PC2 (17%)
Significant traits
Case vs Control
Histology
Positive ER-status vs. Control
Positive PR-status vs. Control
Negative Her2-status vs. Control
Stage 3 vs. Control
H6N5L2E1
H6N5F1L3
H4N5
H5N4F1E1
H4N4F1E1
H6N5E1
H6N3E1
H6N5F1L2E1
H7N6F2L3E1
H7N6F2L2E1
H7N6F1L2E2
H7N6F2L1E2
H7N6F1L1E3
H7N6F2L2E2
H7N6F1L1E2
H6N5F2L2E1
