# Supplementary figures and images for: Serum N-glycan profiles differ for various breast cancer subtypes
Source: Glycoconj J. 2021 Apr 20;38(3):387–95. doi: 10.1007/s10719-021-10001-3 (PMC8116229; doi:10.1007/s10719-021-10001-3)

Scores plot

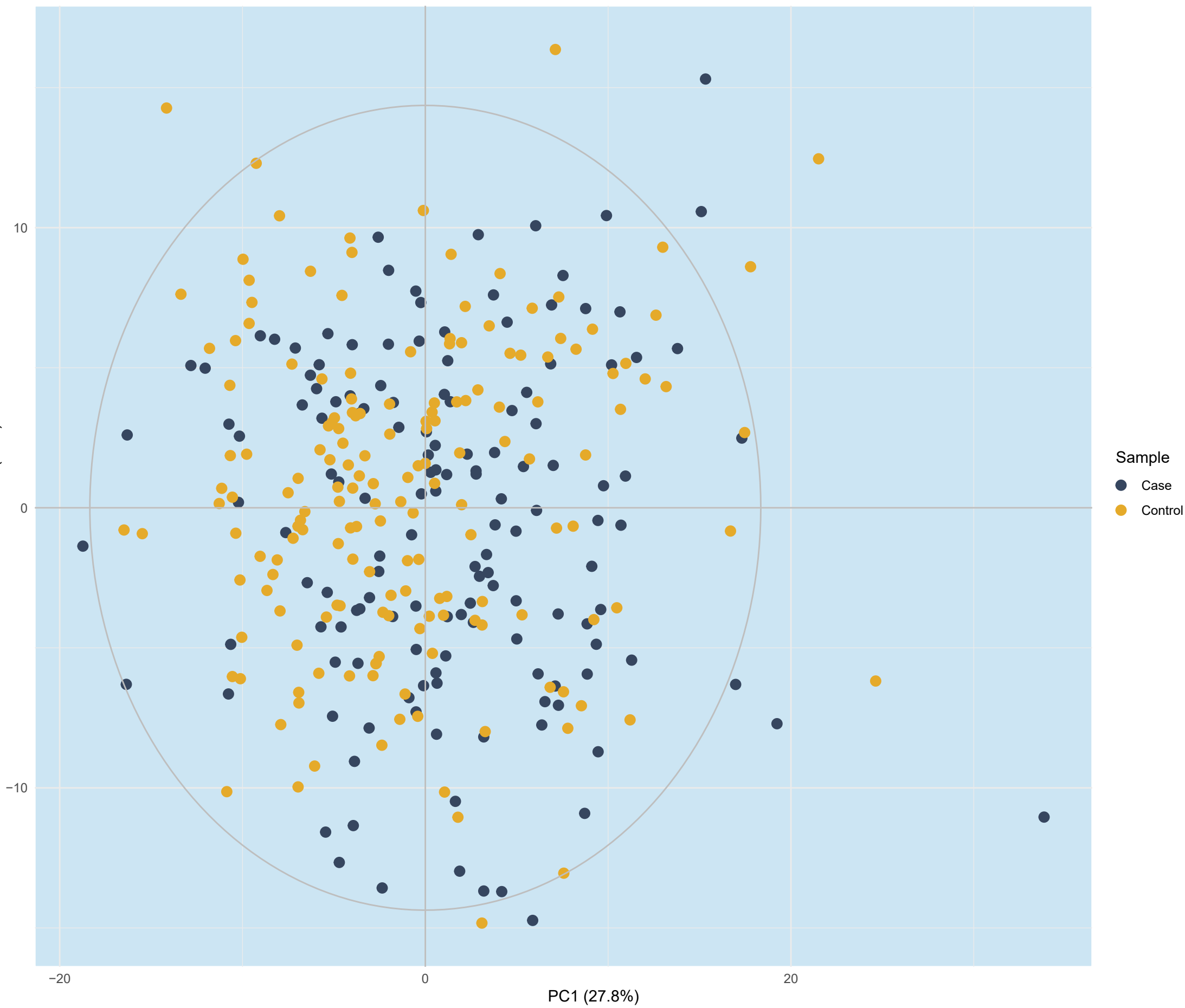

Scores plot

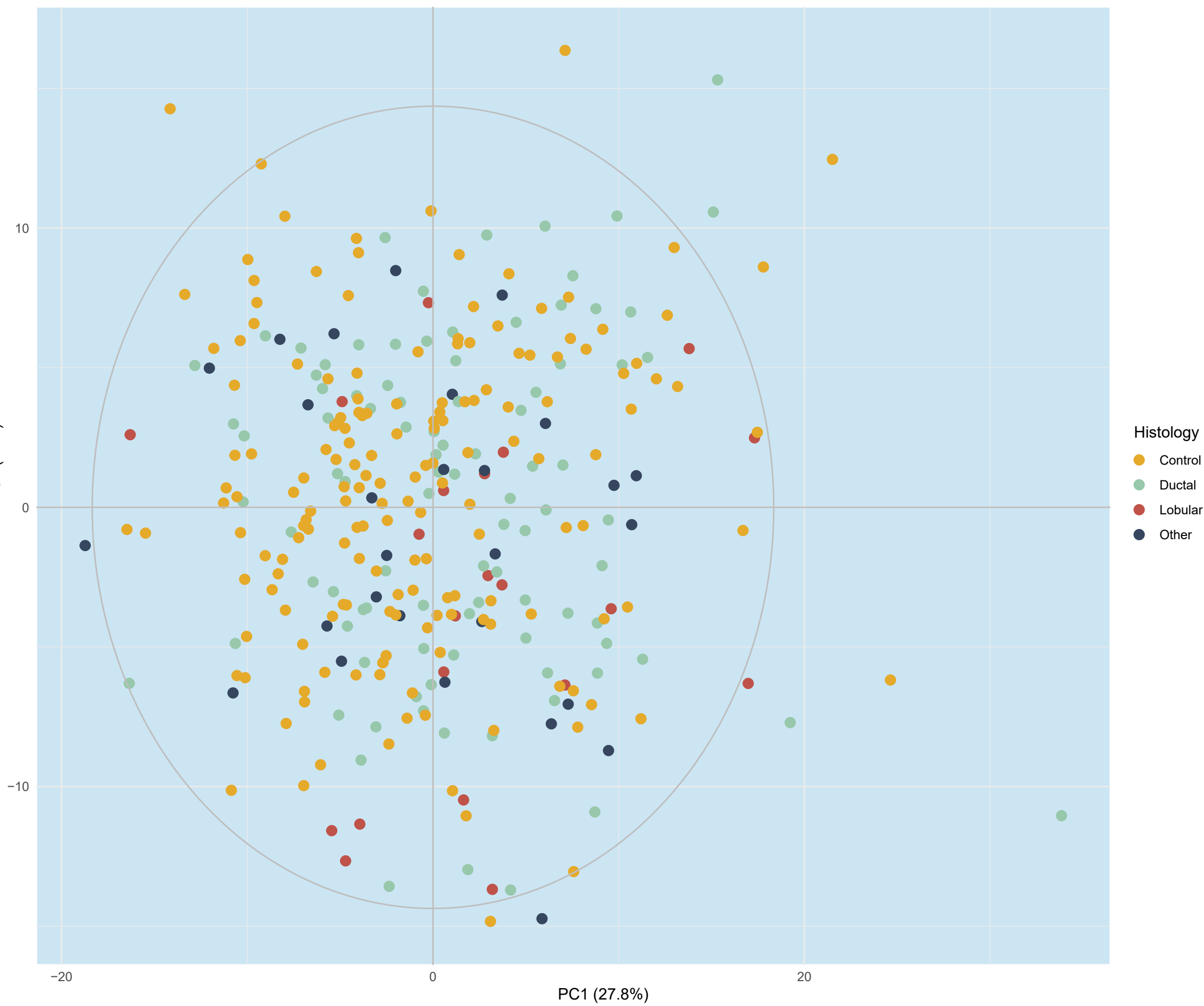

Scores plot

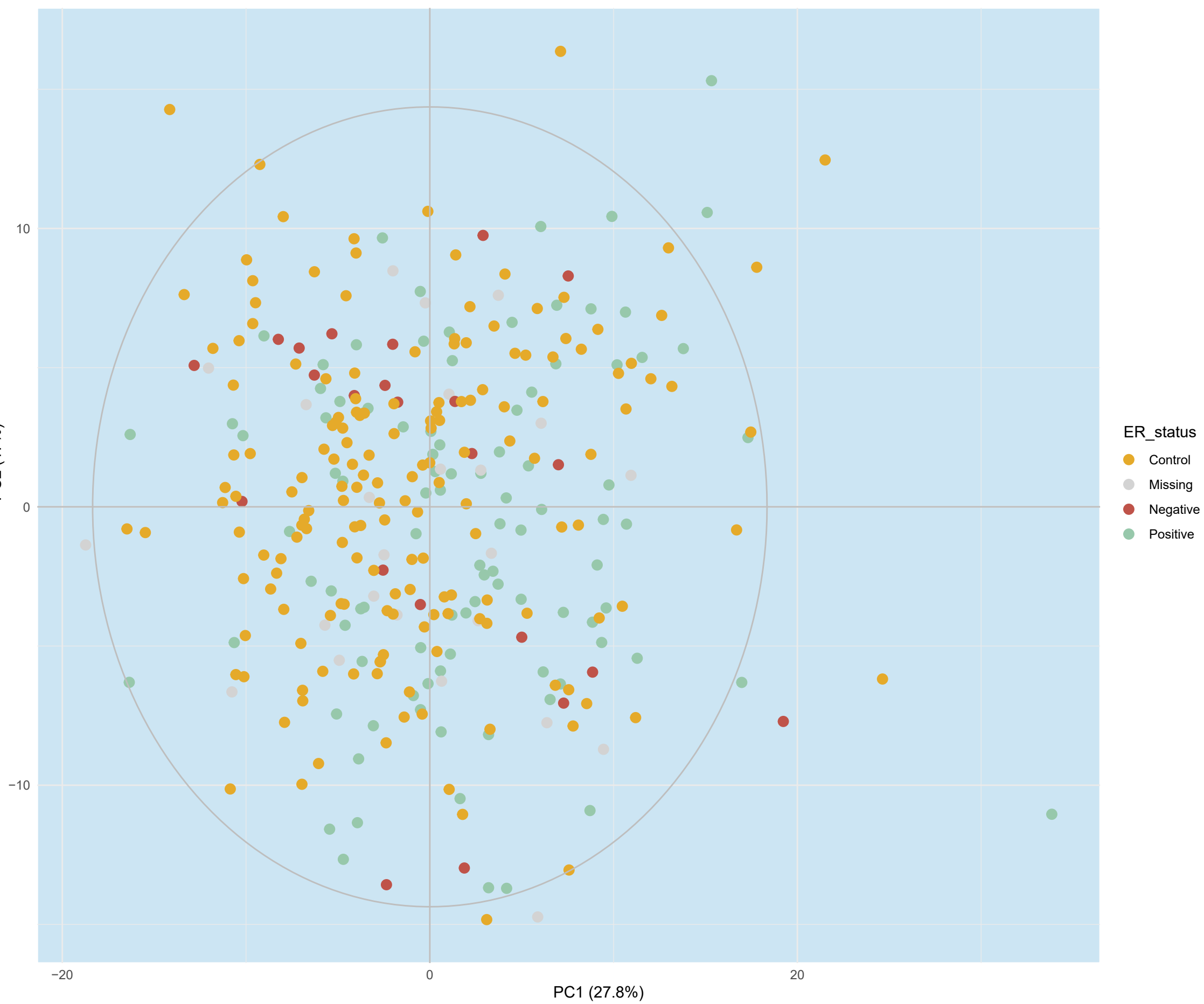

Scores plot

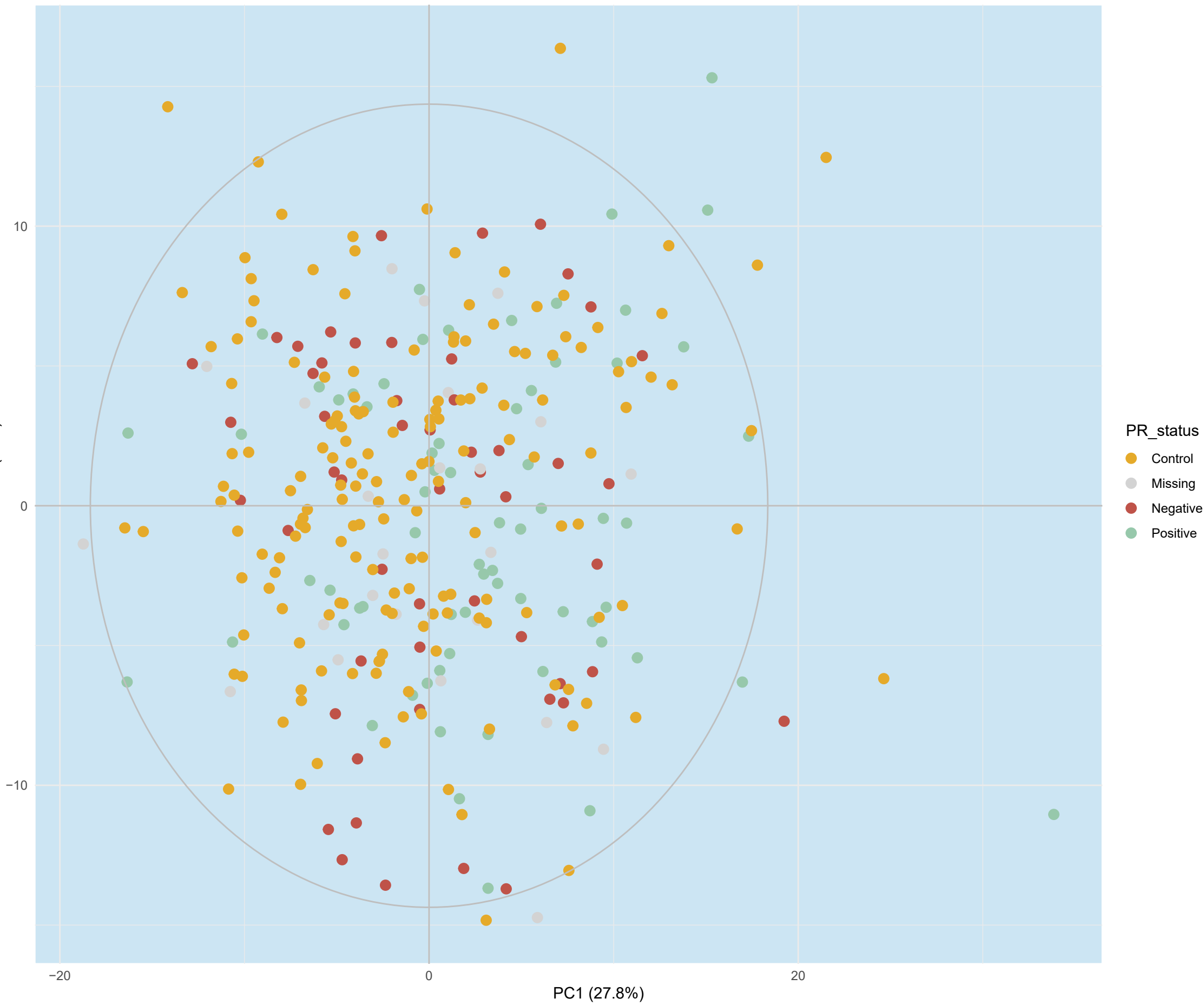

Scores plot

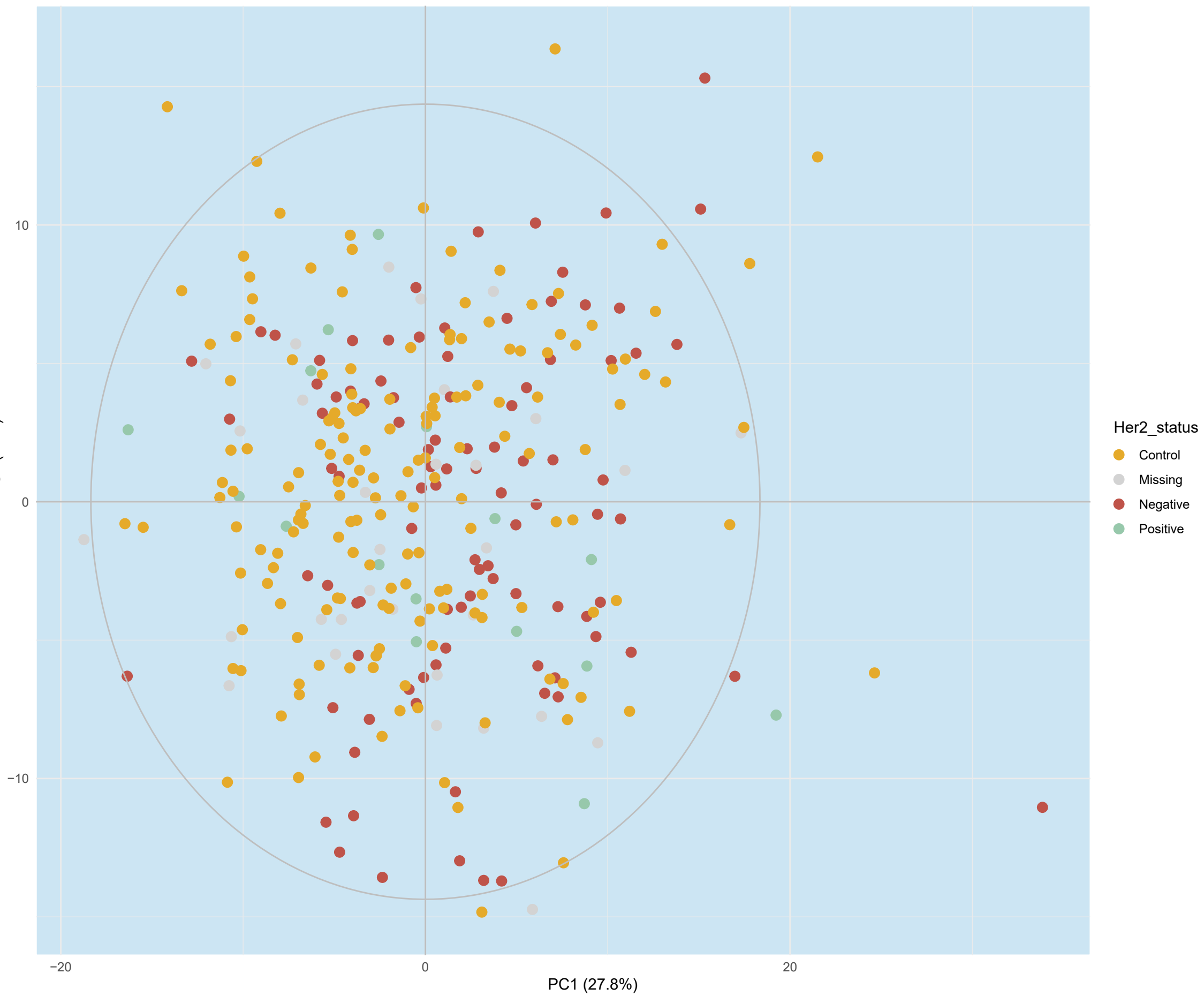

Scores plot

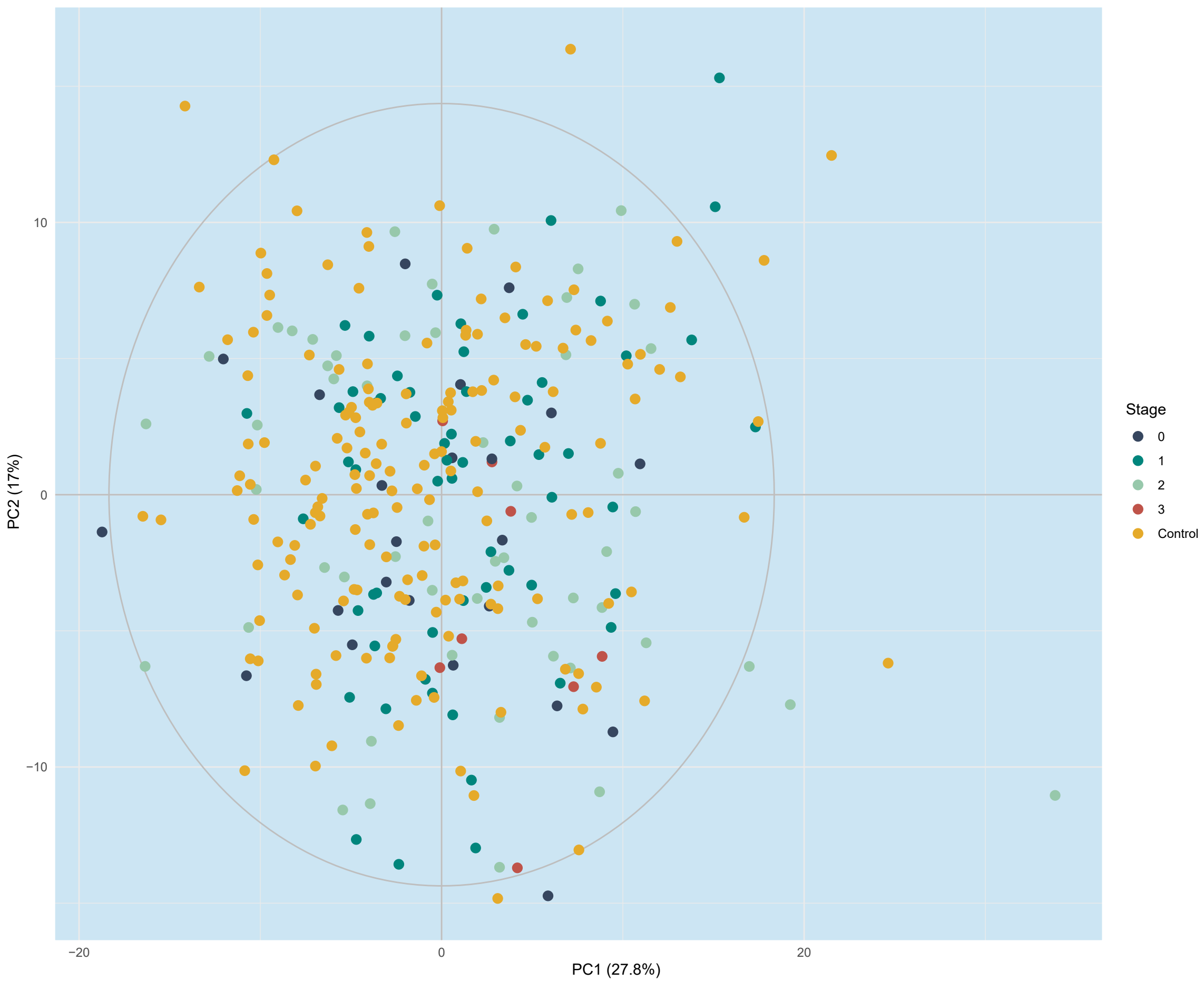

Supplement: Supplementary file 3 — (PDF 503 KB) [file 10719_2021_10001_MOESM3_ESM.pdf]
